# Prevalence of Health Harming Legal Needs of Patients Seeking Care in the Emergency Department

**DOI:** 10.1016/j.acepjo.2025.100062

**Published:** 2025-02-11

**Authors:** Heather Ngai, David Wang, Tim P. Moran, Steve Gottlieb, Candice Priest, Randi N. Smith, Dennis Hsieh, Margaret Samuels-Kalow, Amy Zeidan

**Affiliations:** 1 Department of Emergency Medicine, Harbor-UCLA Medical Center, Torrance, California, USA; 2 Department of Internal Medicine, Emory University School of Medicine, Atlanta, Georgia, USA; 3 Department of Emergency Medicine, Emory University School of Medicine, Atlanta, Georgia, USA; 4 Atlanta Legal Aid Society, Atlanta, Georgia, USA; 5 Department of Surgery, Emory University School of Medicine, Atlanta, Georgia, USA; 6 Central California Alliance for Health, Merced, California, USA; 7 Department of Emergency Medicine, Massachusetts General Hospital, Harvard Medical School, Boston, Massachusetts, USA; 8 Department of Emergency Medicine, Emory University School of Medicine, Atlanta, Georgia, USA

**Keywords:** social determinants of health, health inequities, health-harming legal needs, unmet legal needs

## Abstract

**Objectives:**

Patients seeking care in the emergency department (ED) often have unmet social needs that impact their health and health outcomes. Medical–legal partnerships (MLPs) have been shown to positively impact social and legal needs of patients yet few ED–MLPs exist. The goal of this study was to explore the prevalence of health-harming legal needs (HHLNs) that could be addressed through an ED–MLP.

**Methods:**

We conducted a survey study of a convenience sample of patients seeking care in one ED located in the Southeastern United States over a 6-month period. Research assistants conducted a HHLNs survey following the income/insurance, housing and utilities, education and employment, legal status, personal and family stability (I-HELP) framework with eligible consenting participants. Results were analyzed using descriptive statistics.

**Results:**

Of the total 205 participants, almost all the participants reported one or more HHLNs (n = 187, 91.7%) with many reporting 2 or more overlapping HHLNs (n = 152, 74.1%). The most common HHLNs reported were financial issues, access to benefits, and access to government insurance coverage or benefits. Many participants had concerns about housing and food insecurity.

**Conclusion:**

Our results demonstrate that patients seeking care in the ED had multiple overlapping social and legal needs that could be addressed through an MLP. As EDs increasingly utilize social screeners to identify unmet needs, inclusion of HHLNs would be an important addition to expand resources and interventions available to patients.

**The Bottom Line**

In this study, we explored the prevalence of unmet legal needs that may directly and negatively impact an individual’s health, also known as health-harming legal needs (HHLNs). We found that 91% of patients had at least 1 HHLN and 74% had more than 1 HHLN. Medical–legal partnerships (MLPs) may be an important intervention to consider in emergency departments (EDs) to address unmet legal needs. MLPs have shown to be a promising intervention for addressing adverse social determinants of health and health outcomes yet few ED–MLPs exist.

## 1 Introduction

### 1.1 Background

The impact of unmet social needs and adverse social determinants of health (SDoH) on health outcomes is well documented.^1^^,^^2^ Patients who seek medical care in the emergency department (ED) often have high rates of housing instability, food insecurity, financial challenges, and other unmet social needs.^3^ The extent to which these arise from an unmet legal need and require a legal solution is unknown despite their significant overlap with social needs (see Table 1).^4^^,^^5^ Although ED-based screening for social needs has increased, current strategies do not yet account for legal needs or interventions.^6^^,^^7^ Without considering targeted assistance for legal needs, a social intervention only may not address the unmet need or only partly address it. Although many EDs now incorporate social workers and patient navigators as key components of the health care team, few incorporate legal assistance through medical–legal partnerships (MLPs).^8^^,^^9^

**Table 1:** Relationship between SDoH, social needs, health-harming legal needs and legal solutions.

SDoH^a^	I-HELP health-harming legal needs framework	Health leads social need domain^b^	Health-harming legal need solution
1. Economic stability 2. Health care access and quality	Income and insurance	1. Financial resource strain 2. Food insecurity	• Bankruptcy filings and other collections cessation advocacy • Public benefit denial appeals: SNAP, SSI, SSDI, Medicaid, WIC, and victims’ compensation • Medicaid, Medicare, and private insurance coverage appeals
1. Neighborhood and the built environment	Housing and utilities	1. Housing instability 2. Utility need	• Eviction and foreclosure defense • Lawsuits to address unhealthy and unsafe housing conditions, ie, pests, mold, lead paint, repairs ignored) • Accommodations/accessibility requests • Protection against housing subsidy termination and utility shutoffs
1. Education access and quality 2. Economic stability	Education and employment	1. Employment 2. Education	• Employment discrimination lawsuits • Advocacy to obtain and ensure the proper administration of an individualized education plan • Legal representation in school disciplinary proceedings
1. Economic stability 2. Social and community context	Legal status	N/A	• Veteran discharge status advocacy • Consumer protection advocacy to clear credit history • Immigration law representation
1. Neighborhood and built environment 2. Social and community context	Personal and family stability	1. Interpersonal safety need	• Protective orders • Divorce, child custody, and support • Health care directive, power of attorney, or will

I-HELP, Income and insurance (i), housing and utilities (H), education and employment (E), legal status (L), and personal and family stability (P); SdoH, social determinants of health; SNAP, Supplemental Nutrition Assistance Program; SSDI, Social Security Disability Insurance; SSI, supplemental security income; WIC, women, infants, and children.

a SdoH are a “wider set of forces and systems shaping the conditions of daily life.”^4^ SDoH operate at several structural levels (economics, social policies, political systems, etc.) and directly and indirectly impacts an individual’s health. Social risk is an “individual-level adverse SDoH” (eg, housing instability) but differs from social needs as social needs are those needs that patients explicitly identify as priority.

b Health leads social needs screening Toolkit provides several social needs domains to consider in health care settings.^5^

### 1.2 Importance

An MLP is an interdisciplinary collaboration between health care and legal professionals in which a civil attorney is embedded in a health care setting as part of the medical team.^10^ The attorney helps health care professionals address legal needs that directly impact an individual’s health, also known as health-harming legal needs (HHLNs). These HHLNs can be drivers of ED encounters and are described using the Income/insurance, housing and utilities, education and employment, legal status, personal and family stability (I-HELP) framework.^11^^,^^12^ MLPs have shown to be a promising intervention for addressing SDoH and health outcomes.^11^^,^^13^ MLPs have demonstrated improved health outcomes for patients with asthma, diabetes, HIV, and sickle cell disease. They have also been effective at reducing ED visits and hospitalizations, improving housing stability and maintenance of utilities, and providing financial benefits for patients and health systems.^9^^,^^14^ Thus, the addition of a lawyer to a multidisciplinary team addressing social and legal needs of Patients in the ED would be an important and distinct resource to target specific legal needs (eg, eviction proceedings, landlord–tenant issues). For example, one study demonstrated an increase in ED visits for asthma exacerbations 1 year prior to a failed housing inspection.^15^ With the assistance of an MLP, substandard housing conditions could be addressed in real time, preventing future exacerbations and potential housing insecurity.

### 1.3 Goals of This Investigation

The purpose of this study was to explore the prevalence of HHLNs of Patients in the ED and identify the most common types of HHLNs that could be addressed through an MLP.

## 2 Methods

### 2.1 Study Design and Setting

This was a cross-sectional HHLNs survey of a convenience sample of patients seeking care in the ED from November 2020 to April 2021. The study was conducted at a large, Southeastern United States urban, academic, safety net, level I trauma and emergency care with over 150,000 annual visits. Over 40% of the hospital’s patients are uninsured and those with insurance are likely to have government sponsored insurance. Over 80% of patients identify as Black or African American and approximately 10% of patients speak a language other than English. Research assistants (RAs) based in the ED were available to enroll patients during a 20-hour period (7 am-3 am) 7 days per week and enrolled throughout the ED (eg, in low acuity, high acuity, trauma). RAs consented patients and verbally conducted the HHLNs survey with patients in their preferred language with the assistance of a video interpreter service if needed. All responses to survey questions were entered directly into Redcap by RAs. Participant demographics were not collected to reduce confidentiality-related barriers to participation. This study was approved by the University Institutional Review Board and Hospital’s Office of Research Administration.

### 2.2 Selection of Subjects

Eligible participants included all patients at least 18 years of age who presented to the ED. Patients were excluded if they lacked the capacity to consent (eg, intoxication, altered mental status) or were seriously ill. Patients were approached after being placed in an assigned room/hallway area.

### 2.3 Measures

The HHLNs survey tool was designed in collaboration with a local MLP with extensive experience operating at the local Children’s Hospital. Although no validated HHLN survey exists, the survey questions included questions from pre-existing MLP survey tools (following I-HELP categories) and were refined in partnership with the local MLP. Refinement of the survey included revisions that reflected the local legal context and legal support available in the city where the study was conducted. The survey was written at a sixth-grade reading level to account for varying levels of literacy and took approximately 10 to 15 minutes to complete (without an interpreter). The survey included 19 questions specific to financial issues, access to benefits, housing conditions, end-of-life planning, guardianship and custody, intimate partner violence, and immigration status; all of which have a legal intervention. For participants who inquired about legal services, local resources were provided.

### 2.4 Data Analysis

Analyses were primarily descriptive in nature. HHLNs were described using frequencies and percentages. Analyses were conducted with R.^16^

## 3 Results

A total of 205 participants completed the survey. Most of the participants spoke English, and a Spanish video interpreter was used for 7 participants (3.4%). Almost all participants reported one or more HHLNs (n = 187, 91.2%) with 74.1% of participants (n = 152) reporting 2 or more HHLNs (Table 2).

**Table 2:** Participant responses to health-harming legal needs survey.

Health-harming legal need	N (Total N = 205)	Percent
Does anyone in your household have concerns about financial issues (including bankruptcy, collections, paying for medications, paying for medical bills)	88	42.9
Does anyone in your household have concerns about access to benefits (including SSI, SSDI, WIC benefits)	62	30.2
Have you recently lost your job or are you at risk of losing your job	44	21.5
What is your housing situation today		
I do not have housing	40	19.5
I have housing today but am worried about losing it in the future	27	13.2
Do you have concerns about the condition of your housing	45	22
Do you have concerns about eviction or foreclosure	28	13.7
Within the past 12 months, have you worried that your food would run out before you got money to buy more		
Yes, often	33	16.1
Yes, sometimes	66	32.2
Have you applied for food stamps in the past		
Yes	97	47.3
I have been denied or terminated	13	6.3
Do you and/or does someone in your household have concerns about access to health care, specifically government insurance coverage or benefits (Medicaid/Medicare)	60	29.3
Do you have a child that has difficulty learning in school	19	9.3
If you have a child that has trouble learning in school, does your child have a helpful learning plan in place that the school follows		
Yes, they have a plan that is followed	9	4.4
Yes, they have a plan but it is not followed	4	2
Do you and/or does someone in your household have concerns about their immigration status	12	5.9
Do you and/or does someone in your household have concerns about wills, advance directives, power of attorney, or any other form of end-of-life planning	27	13.2
Do you and/or does someone in your household have concerns about issues related to custody, guardianship, child support, or divorce	28	13.7
Do you and/or does someone in your household have concerns about domestic violence	11	5.4
If a lawyer were available to you for free, would you want their help with any of the above issues	161	78.5
Are there any other legal issues you are experiencing that you would want to talk with a lawyer about	25	12.2
No. of HHLNs		
0	18	8.8
1	35	17.1
2 or more	152	74.1

HHLN, health-harming legal need; SSDI, Social Security Disability Insurance; SSI, supplemental security income; WIC, women, infants, and children.

Most participants (n = 161) noted that they would want help from a lawyer if one were available to them for free. The most common HHLN was financial issues (bankruptcy, collections, paying for medications, paying for medical bills), which was reported by 42.9% of participants (n = 88), followed by access to benefits reported by 30.2% (n = 62) and access to government insurance coverage or benefits reported by 29.3% (n = 60). When asked about housing-related concerns, 19.5% of participants (n = 40) reported no housing, 13.2% (n = 27) reported concerns about losing housing and 13.7% (n = 28) reported concerns about eviction or foreclosure. Nearly a quarter of participants (22.0%, n = 45) reported concerns about the condition of their housing. With regard to employment, 21.5% (n = 44) reported recently losing their job or at risk of losing their job and 8.3% (n = 17) reported applying for unemployment benefits. Nearly half of respondents (48.5%, n = 99) reported that they worried often or sometimes about running out of food before having enough money to buy more in the past 12 months and 47.3% (n = 97) applied for food stamps in the past.

## 4 Limitations

Our study has several limitations. This study was completed at one hospital, which limits generalizability. However, it may be generalizable to hospitals that serve as a safety net and/or care for low-income populations. Our study had a small sample size, and we did not employ randomization or other measures to address selection bias. However, the purpose of this study was to understand the prevalence of HHLNs and most common HHLN to support the development of a legal intervention, an ED-based MLP. The results indicate a high prevalence of HHLNs, which is not surprising to the authors given the patient population at the study site, a safety net hospital where the patient population has several documented disparities related to insurance access, housing, food insecurity, and educational attainment. The HHLNs survey that was used included general legal needs tailored to the local context in which a local MLP could directly intervene. These may be different depending on the city- and state-specific policies that impact patients and the legal resources that are available. The HHLNs survey has questions that may be leading, but all questions were included because they have a potential legal intervention. Finally, the percentage of non–English-speaking patients at the study site is around 10% suggesting under enrollment of this population. Thus we may not have accurately captured the HHLNs of non–English-speaking patients.

## 5 Discussion

Our study revealed that the majority of participants had at least 1 HHLN and a large subset of patients had multiple overlapping HHLNs. The most common needs were related to financial challenges (bankruptcy, collections, paying for medications, paying for medical bills), access to public benefits, and housing. A few prior studies have evaluated HHLN among Patients in the ED.

Although there are over 450 MLPs operating in health care organizations in the United States, there are very few ED–MLPs in which a lawyer or MLP works directly in the ED.^9^ Most MLPs are located in primary care settings and children’s hospitals (that provide primary care) where a legal office or legal professional is incorporated at the clinical site.^9^ Some models utilize off-site legal services or a combination of on- and off-site services, providing referrals to law school clinics, law firms, and legal aid organizations. Several existing models target populations with specific diagnoses (eg, asthma, sickle cell, diabetes, HIV). The most common legal needs provided by existing MLPs are related to housing, utilities, income, personal/family stability, and health insurance. An ED–MLP could be a critical location as many patients in the ED often have barriers to routine care where most existing MLPs operate. Evaluation of MLPs have demonstrated success in addressing SDoH, improving health and health outcomes, and had been shown to have positive financial impacts for both participants and health systems.^11^^,^^14^^,^^17^, ^18^, ^19^, ^20^ The implementation of ED–MLPs could serve as an important component of the existing framework for addressing unmet social needs in the ED. As EDs expand their utilization of social screeners and subsequent interventions to address social needs, it is important to consider screening tools that incorporate both social needs and HHLNs and the process for legal referrals. One existing model, the Highland Health Advocates, demonstrated success in connecting patients with legal and social resources.^21^ In this model, an ED-based help desk was implemented and staffed by undergraduate volunteers who were supervised by social workers within an existing MLP. There are several MLP models that could be implemented in a variety of ED settings as described in the National Center for MLP toolkit.^22^ Legal professionals could work directly with existing programs (eg, social workers, community health workers, clinicians) to expand the continuum of services available, providing education to health teams about legal options, advising patients directly, and/or providing legal representation.^22^ Referrals could be focused on a narrow list of HHLNs (eg, housing, benefits) or an expanded list depending on the local needs and MLP capacity. We recognize that not all EDs may be able to employ a lawyer because of resource constraints. However, there are several models that could be considered in resource-limited EDs, specifically those that partner with and refer to local legal societies with health law units (every state has one or more of these pro bono legal organizations).

Patients seeking care in our ED had a high prevalence of HHLNs. The addition of an ED–MLP to existing services may be an important intervention to address unmet needs. These results apply to our ED and may not be generalizable to other settings. Further research is warranted to understand the prevalence of HHLNs in other EDs, best practices for implementation and assessment of ED-based MLPs, and more specifically their impact on ED and hospital utilization, health status, and health outcomes.

## Author Contributions

H.N. interpreted data, drafted and revised the manuscript. D.W. assisted with study design, acquired data, and analyzed and interpreted data. T.M. assisted with study design, analyzed and interpreted data, and revised the manuscript. S.G.,C.P., R.N.S., and D.H. assisted with study design, interpreted data, revised the manuscript. M.S.K. interpreted data and revised the manuscript. A.Z. conceived and designed study, interpreted data, critically revised the manuscript.

## Funding and Support

By *JACEP Open* policy, all authors are required to disclose any and all commercial, financial, and other relationships in any way related to the subject of this article as per ICMJE conflict of interest guidelines (see www.icmje.org). The authors have stated that no such relationships exist.

## Conflict of Interest

All authors have affirmed they have no conflicts of interest to declare.
