# Supplementary material for: Prevalence of Health Harming Legal Needs of Patients Seeking Care in the Emergency Department
Source: J Am Coll Emerg Physicians Open. 2025 Feb 11;6(2):100062. doi: 10.1016/j.acepjo.2025.100062 (PMC11868935; doi:10.1016/j.acepjo.2025.100062)
Supplement: Supplementary Material [file mmc1.pdf]

Legal Needs Survey - Emergency Department

Please respond to the questions below for statements that apply to YOU or ANYONE in your household.

---

1) If the participant requires interpreter services, please follow the instructions for interpretation below: ________________________________

Phone Interpretation
· call extension: 5-9626 option "3" or 404-616-9626 option "3"
· Select the Language
· The Language Line rep will ask for Facility, Department, and Medical Record #

o Facility: Grady Hospital
o Department: "Health Harming Legal Study"
o MRN: Enter the MRN on the patient's bracelet (or say unknown if you don't have it)

Video Interpretation
· Using VRI iPad, select the yellow Language Line app
· Select the Language
· The Language Line rep will ask for Facility, Department, and Medical Record #

o Facility: Grady Hospital
o Department: "Health Harming Legal Study"
o MRN: Enter the MRN on the patient's bracelet (or say unknown if you don't have it)

2) VERBAL CONSENT:

We are completing a survey study to understand the legal needs of patients who seek care in the Emergency Department.

We would like to better understand what common legal challenges you face and how that impacts your health.

This study will take about 10-15 minutes to complete. Would you be interested in completing the survey?

The risk to you as a participant is minimal. We will do everything in our power to keep your information and responses confidential.

No information about you (ex name, dater of birth) will be included in the survey, only your responses.

There is no direct benefit to you for participating in the study. However, this information may help us improve the services available to our patients in the future.

Your survey responses will be recorded and may be used in future presentations and publications.

This will not include any information that will identify you personally.

Your participation is completely voluntary. You may choose to discontinue or stop at any time.

Do you have any questions?
Do you agree to participate in the survey?

- ◯ Yes
- ◯ No

3) Date of Assessment

____

4) Does anyone in your household have concerns about Financial Issues (including bankruptcy, collections, paying for medications, paying for medical bills)?

- ☐ Yes
- ☐ No
- ☐ Unsure

5) Does anyone in your household have concerns about Access to Benefits (including supplemental security income (SSI), social security disability insurance (SSDI), Women Infants, and Children (WIC) benefits)?

- ☐ yes
- ☐ no
- ☐ unsure

6) Have you recently lost your job or are you at risk of losing your job?

- ☐ yes
- ☐ no

7) If you recently lost your job, have you applied for unemployment benefits?

- ☐ yes
- ☐ no
- ☐ unsure

8) What is your housing situation today?

- ☐ I have stable housing
- ☐ I do not have housing
- ☐ I have housing today but am worried about losing it in the future

9) Do you have concerns about the condition of your housing (example water leaks, bugs, mold lead paint)?
- [ ] yes
- [ ] no
- [ ] unsure
- [ ] not applicable

10) Do you have concerns about eviction or foreclosure?
- [ ] yes
- [ ] no
- [ ] unsure
- [ ] not applicable

11) Within the past 12 months, have you worried that your food would run out before you got money to buy more?
- [ ] yes, often
- [ ] yes, sometimes
- [ ] no, never

12) Have you applied for food stamps in the past?
- [ ] yes
- [ ] no
- [ ] I have been denied or terminated
- [ ] unsure

13) Do you and/or does someone in your household have concerns about access to healthcare, specifically government insurance coverage or benefits (Medicaid/Medicare)?
- [ ] yes
- [ ] no
- [ ] unsure

14) Do you have a child that has difficulty learning in school?
- [ ] yes
- [ ] no
- [ ] unsure

15) If you have a child that has trouble learning in school, does your child have a helpful learning plan in place that the school follows?
- [ ] yes they have a plan that is followed
- [ ] yes they have a plan but it is not followed
- [ ] no they don't have a plan but they need one
- [ ] no they don't have a plan and don't need one
- [ ] unsure

16) Do you and/or does someone in your household have concerns about their immigration status?
- [ ] yes
- [ ] no
- [ ] unsure

17) Do you and/or does someone in your household have concerns about wills, advance directives, power of attorney, or any other form of end of life planning?
- [ ] yes
- [ ] no
- [ ] unsure

18) Do you and/or does someone in your household have concerns about issues related to custody, guardianship, child support or divorce?
- [ ] yes
- [ ] no
- [ ] unsure

19) Do you and/or does someone in your household have concerns about domestic violence?
- [ ] yes
- [ ] no
- [ ] unsure

20) If a lawyer were available to you for free, would you want their help with any of the above issues?
- [ ] yes
- [ ] no
- [ ] unsure

21) Are there any other legal issues you are experiencing that you would want to talk with a lawyer about?
- [ ] yes
- [ ] no
- [ ] unsure

22) If you are experiencing other legal issues not listed above, what are they? ______________________________

23) For the ASSET team member, was an interpreter used for this survey?
- ◯ Yes
- ◯ No

24) If an interpreter was used, what language was requested? ______________________________
